# Supplementary material for: Clinical Research in Chronic Lymphocytic Leukemia in Pakistan; A Systematic Review
Source: Medicina (Kaunas). 2023 Aug 17;59(8):1483. doi: 10.3390/medicina59081483 (PMC10456454; doi:10.3390/medicina59081483)
Supplement: Supplementary file 1 [file medicina-59-01483-s001.zip › Supplemental index I.pdf]

Supplemental Index S1

Database	Search Strategy	Articles
PubMed	(Lymphoma, Lymphoplasmacytoid, CLL OR Lymphoma, Small-Cell OR Lymphoma, Small Cell OR Lymphomas, Small-Cell OR Lymphoplasmacytoid Lymphoma, CLL OR CLL Lymphoplasmacytoid Lymphomas OR Lymphoma, CLL Lymphoplasmacytoid OR Small-Cell Lymphoma OR Small Cell Lymphoma OR Lymphoblastic Leukemia, Chronic OR Chronic Lymphoblastic Leukemias OR Lymphocytic Leukemia, Chronic OR Chronic Lymphocytic Leukemias OR Lymphocytic Leukemia, Chronic, B Cell OR Lymphocytic Leukemia, Chronic, B-Cell OR Lymphocytic Lymphoma OR Lymphocytic Lymphomas OR Lymphocytic Lymphoma, OR B Cell Chronic Lymphocytic Leukemia OR B-Lymphocytic Leukemia, Chronic OR B Lymphocytic Leukemia, Chronic OR B-Lymphocytic Leukemias, Chronic OR Chronic B-Lymphocytic Leukemia OR Chronic B-Lymphocytic Leukemias OR Chronic Lymphocytic Leukemia OR Leukemia, B Cell, Chronic OR Leukemia, B-Cell, Chronic OR Chronic Lymphatic Leukemia OR Chronic Lymphatic Leukemias OR Leukemias, Chronic Lymphatic OR Lymphoma, Small	69

	Lymphocytic OR Lymphocytic Lymphoma, Small OR Lymphomas, Small Lymphocytic OR Small Lymphocytic Lymphoma OR Small Lymphocytic Lymphomas OR Lymphoma, Small Lymphocytic, Plasmacytoid) AND (Pakistan OR Islamic Republic of Pakistan)	
Google Scholar	Chronic lymphocytic leukemia; Pakistan	94
Clinicaltrials.gov	Conditions or disease: Chronic lymphocytic leukemia<br>Country: Pakistan	0

Date when search performed: January 14th, 2022

Place of search: Rochester, New York, United States
